# Putting a human in the loop: Increasing uptake, but decreasing accuracy of automated decision-making

**DOI:** 10.1371/journal.pone.0298037

**Published:** 2024-02-09

**Authors:** Daniela Sele, Marina Chugunova

**Affiliations:** 1 Center for Law & Economics, ETH Zurich, Zurich, Switzerland; 2 Max Planck Institute for Innovation and Competition, Munich, Germany; Lingnan University, The University of HongKong, HONG KONG

## Abstract

Automated decision-making gains traction, prompting discussions on regulation with calls for human oversight. Understanding how human involvement affects the acceptance of algorithmic recommendations and the accuracy of resulting decisions is vital. In an online experiment (N = 292), for a prediction task, participants choose a recommendation stemming either from an algorithm or another participant. In a between-subject design, we varied if the prediction was delegated completely or if the recommendation could be adjusted. 66% of times, participants preferred to delegate the decision to an algorithm over an equally accurate human. The preference for an algorithm increased by 7 percentage points if participants could monitor and adjust the recommendations. Participants followed algorithmic recommendations more closely. Importantly, they were less likely to intervene with the least accurate recommendations. Hence, in our experiment the human-in-the-loop design increases the uptake but decreases the accuracy of the decisions.

## Introduction

Today, algorithms are increasingly used to make decisions of economic and legal importance. Automated decision supports are already used by companies and public institutions for a variety of tasks—from evaluating job applications, to deciding what salary or bonus to offer or even to bail and parole decisions [1–4]. The increased use of such (partially) automated decisions may have significant legal and economic effects and has been accompanied by calls for policies that put a “human in the loop” (e.g., Art. 22 of the EU’s General Data Protection Regulation (GDPR) [5] or Art. 14 of the EU’s draft AI Act [6]). These policies envision a system where a human monitors and interacts with an automated decision support system by both relying on the inputs provided by the system, but also consistently and thoughtfully analyzing them. From a legal perspective, a human monitor is introduced to exercise oversight over the decision-making process to maintain human agency and accountability, provide legal safeguards, or perform quality control [7].

Behavioral research raises concerns about the seamless functioning of such hybrid decision systems and emphasizes that human behavior in them might be systematically different. However, there is a need for further understanding of the exact patterns of the differences [8]. When deciding whether to use the automated decision support system, people were found both averse to using algorithms in decision-making [see *algorithm aversion* in 9, 10, for an overview] and appreciative of them [see *algorithm appreciation* in 11, 12]. When engaging with the algorithmic recommendations, users relied on the automated support too little by not incorporating the recommendations into their decisions [11, 13]—and too much by failing to appropriately correct their mistakes [*automation bias and automation-induced complacency*, see [14, 15], for an overview]. Against the backdrop of the importance of legal and policy discussion, the existing evidence provides little guidance for the role of human agency in interaction with automated decision supports.

In this paper, we consider two research questions: First, we study if moving from a fully automated decision-making system to a human-in-the-loop system increases the preference for using an algorithmic decision support (over a human one). As conjectured in [8], one possible explanation for seemingly inconsistent and conflicting findings is the allocation of decision-making authority between humans and machines in an automated decision-making situation. In this case, introducing a Human-in-the-Loop system can increase the uptake of the automated decision supports. Second, we study if keeping a human in the loop results in effective monitoring of algorithmic decisions. We consider how human monitors engage with recommendations from different sources and if their adjustments improve the accuracy of the decisions. If the monitoring human blindly rubberstamps the recommendations of the system and follows them as “default” decisions [16], the human-in-the-loop might not play the role that policy makers hope for.

Answering these questions is of high applied importance to organizations introducing automated decision-making supports into their work processes or developing them as well as to policy-makers who aim to introduce regulatory safeguards. An important example of such regulatory safeguards that apply to automated decision-making is Art. 22 of the EU GDPR. It prohibits fully delegating a decision to automated means if it produces legal effects on a human decision subject or similarly impacts that individual. The effectiveness of this article is disputed by some commentators, see e.g. [17] or conversely [18]). The draft of the EU’s AI Act further develops the idea that automated decision systems require human oversight. In Art. 14 a specific obligation to human oversight in the development of high-risk AI systems is proposed. The AI act will be the first law on AI by a major regulator and is likely to have a global impact both due to “Brussels effect”—organizations voluntary applying higher standards required by the EU regulation outside of the EU [19]—and setting a legislative precedent for comprehensive AI-specific regulation in other countries [20]. The widely signed Montréal Declaration of Artificial Intelligence [21] also states that “[i]n all areas where a decision that affects a person’s life, quality of life, or reputation must be made, where time and circumstance permit, the final decision must be taken by a human being and that decision should be free and informed” (principle 9.1).

To provide empirical evidence to answer these questions we conduct an online experiment with a prediction task: Participants are asked to predict the performance of a student in a standardized math test based on the student’s profile [as previously used in [22]. To assist the performance prediction, participants are offered an estimate from one of two sources: either from another human participant (generated in a pre-experimental session) or from a statistical model. Participants are informed that both the estimates of the other participants and the model are of equal quality on average. Using a between subject design, we vary if participants *delegate* the prediction fully to the provider of the estimate (Delegation condition) or whether they can *adjust* it before submitting the performance prediction (Human-in-the-Loop condition). In the following, for the sake of brevity we refer to the estimates participants receive from either a human or an algorithm as “recommendation” in both the Delegation and Human-in-the-Loop conditions, although in the Delegation condition participants cannot adjust the “recommendation”.

We find both a general preference for automated decision support, and that this preference increases further when the human principal is allowed to retain some agency over the decision. Indeed, even in the Delegation condition, participants chose to delegate the decision to an algorithm rather than to another human in 66% of cases. As human and algorithmic recommendations were curated to be equally accurate and participants were informed about it, this finding speaks for a preference for an algorithm. This result is in line with several recent papers [e.g., 23, 24] that also do not find algorithm aversion even under full delegation. Allowing participants to adjust the recommendation further significantly increases the likelihood to opt for a recommendation by an algorithm by 11% (7 percentage points). Hence, we find evidence that the retention of human oversight can significantly increase the willingness to use automated decision-making support. In the Human-in-the-Loop condition participants also report feeling more confident in the predictions they submitted regardless of the source of the recommendation.

When investigating how participants engage with the recommendations, we find evidence of automation bias (i.e., of over-reliance on the automated inputs): Participants tend to follow recommendations produced by algorithms more closely than those by humans (although they are almost always identical). In our experimental environment, we also find that the participants’ adjustments decrease the accuracy of the final predictions: Within the Human-in-the-Loop condition, participants appear to particularly struggle to appropriately adjust the recommendations that stem from an algorithm. Predictions submitted following the algorithmic recommendation appear to be (insignificantly) less accurate. Probably most problematically from the perspective of decision quality, the human monitors are less likely to adjust recommendations that contain larger errors as compared to smaller ones regardless of the source. Moreover, the adjustments made to recommendations with larger errors also tend to be smaller. All of these findings raise questions on the effectiveness of policies that propose the retention of a human in the loop to ensure the quality of the decision-making. The vivid discussions around such policies however point to a wish to retain such human oversight. Indeed, as a final result of our experiment (and similar to the general population [25, 26]), the vast majority of participants believe that a human should almost always be put in place to monitor algorithmic decisions.

In summary, the findings of our experiment hence highlight an important trade-off: while the retention of human oversight can increase the uptake of automated decision-making support, it may also decrease the quality of the final decisions. Our experiment demonstrates this in a stylized version of a task where automated recommendations are already widely used, namely admission processes for university or higher schooling placements [e.g., 27]. In the wide range of possible applications of automated recommendations, this trade-off may be resolved differently for each context-specific application. However, the awareness of it may prove useful when considering the legislative framework surrounding automated decision-making; as we will discuss further on.

## Materials and methods

**Fig 1:**
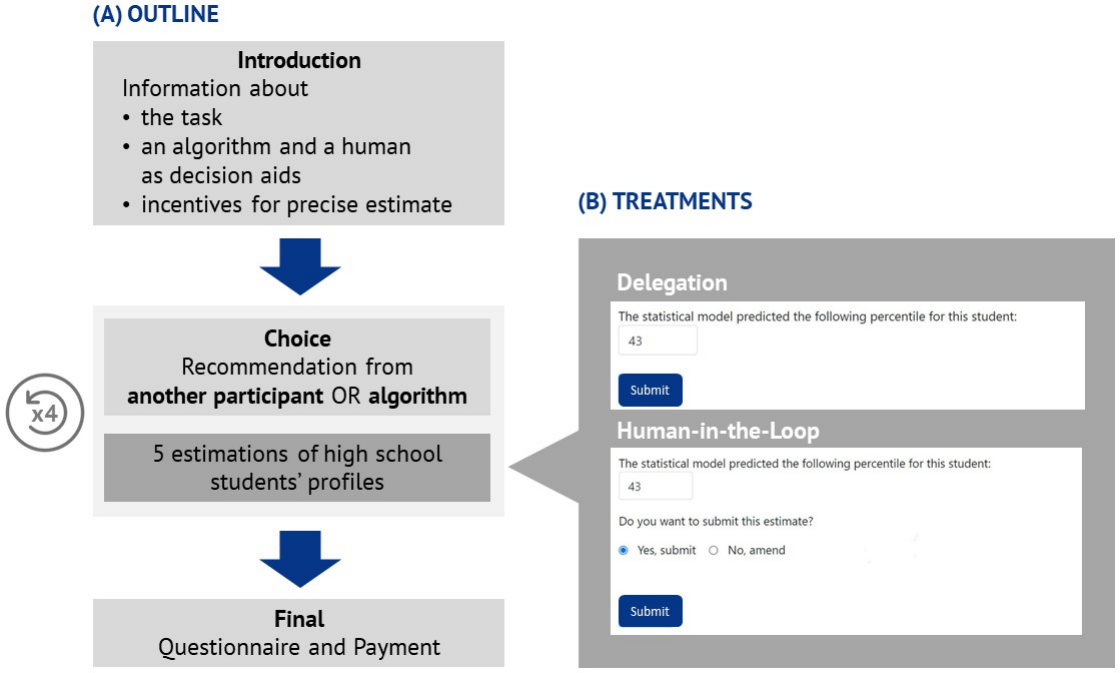
Overview of the experiment and the treatments.

Our online experiment (see Fig 1 for an overview) adopts the task first used in [22], which requires participants to forecast the percentile ranks of U.S. high school students at a nationwide standardized math test based on a short student profile. The profile contains nine characteristics of a student (see S7 and S8 Figs in S1 File): the student’s race, their family’s socio-economic status (in quintiles), their desired occupation at age 30, their self-predicted highest educational degree, the region of the USA they live in, the number of times they took the PSAT, the number of the student’s friends who are not going to college, their favorite subject, and whether the student has taken any AP test. Participants in the study, which took place in Switzerland, where provided with additional explanatory information about all of these items. The task uses real, public data from the U.S. High School Longitudinal Study of 2009.

To make their test performance prediction, participants are offered estimates from one of the two sources: either from *another human participant* or from *a statistical model*. In the further text we use the terms statistical model and algorithm interchangeably. The human estimates were drawn from data generated in a pre-study with US-based participants on Amazon MTurk. The estimates of an algorithm come from a model developed in [22]. When introduced to the task, participants are provided with some basic information about the statistical model and the other participant. The description of both sources was purposefully written to be similar. In particular, participants were informed that both the model and another participants were imperfect, and that both make average mistakes of 15 to 20 percentiles. In more details, participants were informed that the statistical model was designed to forecast the percentile score of a student in the math test, that for its estimations it uses only the information in the displayed profiles and that it is developed in the US by thoughtful analysts. They were also informed that the model’s estimates are off by 15 to 20 percentiles on average. About the other participants they learned that the predictions were made during a pre-study with participants located in the US who also used the information in displayed profiles. They were also informed that the other participants’ estimates are off by 15 to 20 percentiles on average. S9 Fig in S1 File displays a snippet of experimental instructions. In the experiment, the recommendations of the algorithm or the other participant were curated to differ at most by ±2 percentiles, yet participants were only informed about the identical average performance.

Participants could choose if they want to receive the recommendation from an algorithm or another human—rather than choosing between an algorithm’s recommendation and unassisted decision. With this design choice, we deviate from the design of [22] and follow another seminal paper in the field [11]. By looking at the choice between two sources of recommendations we are able to take into account that people generally discount advice relative to their own judgment [28, 29]. Put differently, we aim to investigate the participants’ willingness to use automated rather than human advice without the impact of (potential) over-confidence in their own capabilities.

We conducted two treatments in a between subject design. In the *Delegation* condition, participants fully delegated the decision to the chosen source of a recommendation. That is, the participants could choose between receiving the “recommendation” from either another participant or an algorithm and this “recommendation” was then directly recorded as the participant’s prediction of the student’s performance. In the *Human-in-the-Loop* condition, participants also chose if they want to receive a recommendation from an algorithm or a human but could then either submit this recommendation as is or adjust it. If they chose to adjust the recommendation, they were able to do so without any restrictions (see Fig 1 and S7, S8 Figs in S1 File for example screens).

Participants were asked to make predictions for 20 profiles, which were split into four blocks of five profiles. Before the start of each block, participants were asked to choose if they prefer to receive the recommendations from another participant or from the algorithm. Each participant hence made four choices regarding their preferred source of recommendations. The sequence of profiles was constant for all participants. Importantly, in our experiment we do not provide feedback on the accuracy of the performance predictions during the experiment. This design choice is motivated by the fact that for many applications of algorithmic decision supports, information on the correctness of the prediction is not immediately available.

It is conceivable that participants who select a certain type of the recommendation source might be better able to engage with the recommendations by this type of a source. To explore if selection of the recommendation source affects how its recommendations are used, in the Human-in-the-Loop condition, 40% of times participants were assigned to the source of recommendations different to the one they desired. For instance, if a participant in the Human-in-the-Loop condition chose to receive the recommendations by the algorithm, she would receive the algorithm’s recommendations with *p* = 0.6 and be given the other participant’s recommendations with *p* = 0.4. Participants were informed about this in the beginning of the experiment. They were clearly informed about the source of the estimate, both with an intermittent screen after the choice of the source and consistently on their screens. Due to this feature, we opted for an unequal number of participants per treatment. 292 participants took part in the experiment in total. Upon starting the experiment, participants were assigned to Delegation condition with 40% change, and to Human-in-the-Loop with 60% chance. Therefore, 108 were randomly assigned to the Delegation condition and 184 to the Human-in-the-Loop condition.

### Procedures

Participants were invited using the UAST subject pool jointly used by the University of Zurich and ETH Zurich. The only exclusion criteria was a good command of English and a minimum age of 18. Most of the participants were students at ETH Zurich (54%). The average age was 24 years old. 53% of participants were female. 62% were students in a STEM discipline.

The main experiment was implemented and conducted by the ETH Zurich Decision Science Laboratory (ETH DeSciL) using oTree [30]. Participants received a show-up fee of 5 CHF and were additionally incentivized to make accurate predictions of the high school student’s performance according to the following system: One of the 20 predictions was randomly chosen at the end of the experiment and participants could earn 15 CHF if the performance prediction was within 5 percentiles of the true performance. Participants were paid according to a step function with smaller bonuses paid for less accurate predictions: For every additional 5 percentiles difference between the prediction and true performance of the student the bonus was reduced by additional 3 CHF. Therefore, there was no bonus if the prediction was more than 25 percentiles away from the true performance. Participants earned on average 6.10 CHF as a bonus.

To generate the recommendations from the other human participants that were used in the main experiment, we conducted a pre-study with 200 participants on Amazon MTurk. Participants in this study were U.S. residents and at least 18 years old. There were no further exclusion criteria for the pre-study. Participants made a series of performance predictions and received a bonus payment contingent on the accuracy of one randomly selected performance estimate.

The experiment received prior approval from the ETH Zurich ethics approval board (EK 2021-N-121). Written and informed consent from participants was obtained prior to the experiment. The payment was administered by ETH DeSciL, the researchers had no access to personally identifying information about the participants. The collection of data took place between 30th of September and 11th of October 2021. The data of the pre-study was collected on 21st of September 2021. Participants expressed written informed consent.

## Results

### Preference for the source of recommendation

**Fig 2:**
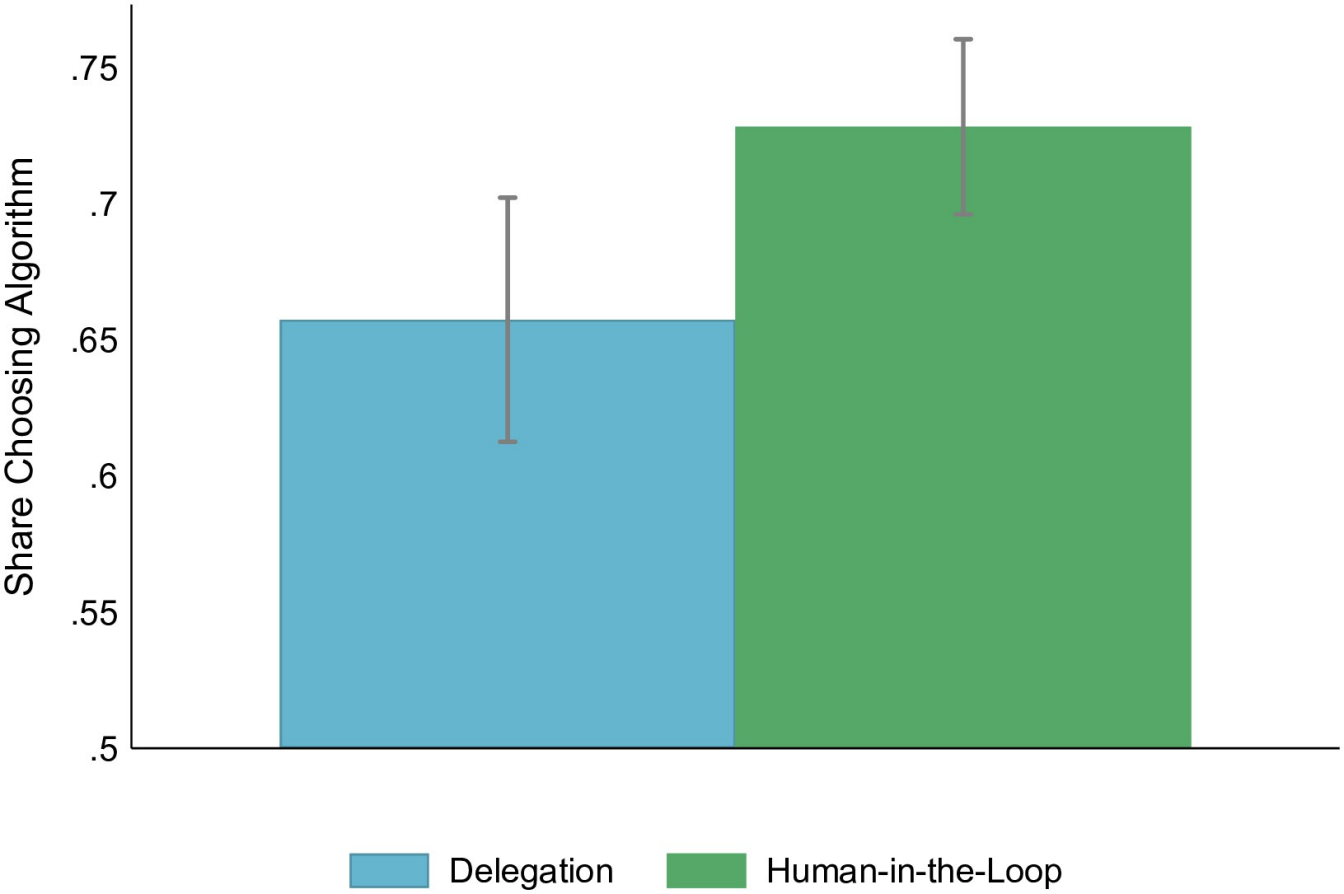
A share of participants who chose to receive a recommendation by an algorithm. 95% confidence intervals.

Our study finds a general preference for algorithmic recommendations. Participants were informed that both recommendations from a human and from an algorithm are on average equally accurate, yet, already in the Delegation condition in 66% of choices participants preferred to receive recommendations from an algorithm. This share is significantly different from 50% which would be expected due to equal performance of the two sources (one-sample test of proportions, *p* < 0.0001). Putting a human in the loop by allowing participants to adjust the received recommendation further increases the preference for using a recommendation from an algorithm by 11% (7pp). The difference between the two treatments is significant (*p* = 0.01). Throughout the text, we report results and significance levels of two-sided t-tests Unless specified otherwise. Fig 2 depicts the shares of participants who chose to receive a recommendation of an algorithm across conditions. The result is reconfirmed by a probit regression controlling for an iteration and a set of demographic characteristics (S1 Table in S1 File). The difference is largely driven by a share of participants who always preferred an algorithm to a human in the Human-in-the-Loop condition (see S6 Fig in S1 File, Kalmogorov-Smirnov test of the equality of distributions, *p* < 0.001).

While the direction of the effect is in line with the results of [22], who find that allowing people to make small corrections to the algorithm increases uptake, the level of the effect and the fact that we find a preference for an algorithm even in the Delegation condition is of interest. Earlier work by [9] had documented widespread algorithm aversion when participants learn that an algorithm can err, leading the authors to suggest that humans may forgive other humans mistakes but remain skeptical to (imperfect) algorithms. Their study also documented that without receiving feedback about the performance of the algorithm, people are either indifferent between receiving the (superior) algorithmic advice or prefer it. In our experiment, the strong preference for an algorithm is striking as unlike in [9] the algorithm and human recommendations were equally good and participants knew that both the statistical model and the other human make mistakes. Yet, an important caveat to this comparison, which only reinforces our result, is that, in the mentioned study participants decided between receiving the algorithmic recommendation or not and not between the source of recommendation. In [11, Experiment 3] where participants choose between the source of recommendation, the share of participants choosing an algorithm in human-in-the-loop treatments is somewhat higher than documented in this paper. [11] find that 88% of participants preferred an algorithm over a human recommendation. The difference to our somewhat lower share might be possibly explained by the almost perfect performance of the algorithm in their study. As we are interested in studying the role of the nature of the recommendation source abstracting from potential performance differences, in the description of the sources in our experimental setting we attempted to convey comparable reliability of the sources. We informed participants about their identical average performance and carefully chose wording to describe them (see S9 Fig in S1 File for the relevant part of the experimental instructions). Nevertheless, readers should be aware that differences in the description of the sources of recommendations might affect the preferences exhibited by participants.

**Result 1**. We find a general preference for receiving recommendations from an algorithm rather than from another human. Moving from a full delegation of the decision to a Human-in-the-Loop system increases the uptake even further.

### Confidence in the decision

In the Human-in-the-Loop condition, participants also report having more confidence in their own estimates than do the participants in the Delegation condition (41 out of 100 in Delegation, 48 out of 100 in Human-in-the-Loop, *p* = 0.008). In general, the result that a Human-in-the-Loop system not only increases uptake but also confidence of the users mirrors the results of [22]. Interestingly, although the participants knew that a human and an algorithm are on average equally accurate, participants of both treatments felt more confident about the recommendations by the algorithm (57 out of 100 as compared to confidence of 46 for human estimates, *p* < 0.0001). This question was asked at the end of the experiment and can only inform us about ex post confidence.

**Result 2**. Participants in the Human-in-the-Loop condition are more confident in the performance predictions they submit.

### Accuracy of the predictions

A crucial question is how accurate, i.e., how close to the true performance of a student, the performance predictions are. As recommendations that participants receive are by design largely identical, the difference in accuracy of final predictions comes from the adjustments the participants can make in the Human-in-the-Loop condition. We measure accuracy as an absolute deviation between a submitted decision and a true performance percentile of a high school student. A smaller deviation between the prediction and the true performance indicate a more accurate prediction. It emerges that the decisions in Delegation condition, where participants could not adjust the received recommendation, are significantly more accurate than in Human-in-the-Loop (17.4 and 18.0, *p* = 0.04, see S1 Fig in S1 File). This result is in line with previous literature that finds that algorithmic forecasts tend to be superior to human ones in a variety of domains [e.g., 11, 31–33]. Recall that in the experiment performance predictions of human participants used as recommendations in the main experiment were curated to be of an equal quality with an algorithm.

**Result 3**. Adjustments made by human monitors in the Human-in-the-Loop condition lead to the average decrease in the decision accuracy.

### Engagement with recommendation

As the overall accuracy decreases in the Human-in-the-Loop condition, it is important to consider how people engage with the recommendations they receive and where inaccurate adjustments of recommendations happen. In the Human-in-the-Loop condition the recommendation was pre-filled. This design choice made it easy for participants not to engage with the recommendation and simply “rubberstamp” it due to default effects [e.g., 16, 34]. If human in the loop blindly accepts recommendations by the algorithm, it raises concerns as to how much they contribute to the desirable features of the system such as maintaining human agency and accountability.

This appears to be less of a concern in our experiment: In 63% of estimations in Human-in-the-Loop treatment participants adjusted the provided recommendation. The average adjustment was 6.9 percentiles (6.7 for recommendations received form an algorithm and 7.1 from another human). To consider if participants are less likely to adjust recommendations received from an algorithm and how the source of the recommendation affects the accuracy of performance predictions, we construct a panel and estimate a fixed effects model. It allows us to focus on the systematic difference in adjustments by the source of recommendation abstracting from individually invariant characteristics. As participants made four choices and in 40% of cases participants were assigned to an alternative recommendation source only 8.7% of participants in the Human-in-the-Loop treatment were exposed only to algorithmic or to human recommendations and had no within subject variation.

We first consider if participants are more likely to adjust the recommendation altogether depending on its source and then if (conditional on being adjusted) the adjustments systematically differ (see Table 1). We fail to find evidence for algorithmic bias on the extensive margin: participants are equally likely to adjust the received recommendations regardless of the source (Table 1, specification 1). Yet, if participants intervene, the size of the adjustment is larger for human recommendations (Table 1, specification 2). Our preferred specification is specification 2 that considers the size of adjustment among the participants who engaged with recommendation and adjusted it. This approach allows to abstract from any differences in the levels of engagement in general, which (although are shown insignificant as per column 1) might bias the results. We report the alternative approach of considering the effect of the source of the recommendation on the size of adjustment including zero adjustments in specification (3) and document a marginally significant effect.

**Table 1:** Column (1) reports estimates of fixed effects logit for a binary outcome (adjust a recommendation or not). The decisions of 6 participants (120 decisions) were omitted because of all positive or all negative outcomes. Columns (2)-(4) report results of fixed effect models on the size of an adjustment and inaccuracy respectively. Column (2) reports coefficients conditional on non-zero adjustment of the recommendation. Column (3) lifts this restriction.

VARIABLES	(1)	(2)	(3)	(4)
	Panel Logit	Fixed Effects	Fixed Effects	Fixed Effects
	Binary: adjusted	Conditional adjustment	Adjustment	Inaccuracy
Another Participant	0.0623	0.868***	0.494*	-0.761
	(0.0810)	(0.330)	(0.289)	(0.700)
Binary: Adjusted				-0.616
				(0.599)
Another participant × Binary: Adjusted				0.437
				(0.857)
Constant		10.50***	6.650***	18.61***
		(0.215)	(0.188)	(0.472)
Observations	3,560	2,320	3,680	3,680
Number of id	178	181	184	184

Standard errors in parentheses

*** p<0.01,

** p<0.05,

* p<0.1

On average the predictions submitted following the recommendation by an algorithm tend to be (insignificantly) less accurate than the recommendation by a human (18.3 and 17.7 percentiles from the true performance, Table 1, specification 4). As provided recommendations for each profile are in most of the cases identical (at most ±2 percentile) and as participants appear to be correcting human recommendations by more, this suggests that participants may particularly struggle with correcting algorithmic recommendations.

**Result 4**. Participants are equally likely to intervene following the recommendations from either source. Yet, in line with automation bias, conditional on intervening the size of the adjustment is smaller for algorithmic recommendations.

### Correcting larger errors

The human monitor in Human-in-the-Loop systems is intended to supervise an algorithm and interfere if decisions it produces are inaccurate. One can argue that the task of the monitor is not to correct every recommendation of the system, but to spot and correct recommendations that contain a large error. To consider if monitors in the Human-in-the-Loop treatment are better at correcting larger as compared to smaller errors, we classify the recommendations that are least accurate (in the top 25% of the absolute deviation from the truth) as larger errors. Following this definition, profiles where the recommendation is at least 26 percentiles away from the true performance were classified as large errors. Our results suggest that human monitors are less likely to intervene when the recommendations are least accurate (64% adjusted a recommendation if it had a small error and 60% if large, *p* = 0.02). If participants decide to intervene, the size of the adjustment is significantly larger for smaller mistakes than for larger ones (adjustment of 11.3 for smaller error and 9.6 for larger ones, *p* < 0.001). The fixed effects and pooled OLS specifications reconfirm this result (see Table 2). We do not find an additional interaction effect: Larger errors are adjusted by less regardless of the source of recommendation. These results suggest that human monitors fail to serve as an “emergency brake” for the recommender system. The results are not sensitive to variations in the definition of “large errors” and hold if treated as continuous. In fact, the larger the error the less likely people are to interfere and the smaller is their adjustment. S2 Table in S1 File reports the estimation without the classification of errors.

**Table 2:** As large errors we classified recommendations with a deviation from the truth in the top 25 percentiles. In model (1) the decisions of 6 participants (120 decisions) were omitted because of all positive or all negative outcomes. In models (2) and (3) we estimate an OLS specification with standard errors clustered at the individual level. (4) and (5) report fixed effects specification.

VARIABLES	(1)	(2)	(3)	(4)	(5)
	Panel Logit	OLS	OLS	Fixed Effects	Fixed Effects
	Binary: Adjusted	Adjustment	Adjustment	Adjustment	Adjustment
Large error	-0.221**	-1.533***	-1.261***	-1.530***	-1.398***
	(0.0859)	(0.298)	(0.431)	(0.308)	(0.437)
Another Participant	0.0639	0.401	0.527	0.502*	0.561*
	(0.0811)	(0.311)	(0.344)	(0.288)	(0.320)
Large error × Another Participant			-0.562		-0.272
			(0.619)		(0.639)
Constant		7.037***	6.978***	6.988***	6.961***
		(0.307)	(0.318)	(0.199)	(0.209)
Observations	3,560	3,680	3,680	3,680	3,680
Number of id	178			184	184

Standard errors in parentheses

*** p<0.01,

** p<0.05,

* p<0.1

We additionally explore what features of the profiles or received recommendations tend to increase the likelihood that the participants intervene and affect their accuracy (see S3 Table in S1 File). We find that seeing low or high recommendations on the profile decreases the likelihood that the participant adjusts the recommendation. As low and high recommendations we classified recommendations that fall in the lowest 25% (below 42 percentiles) and highest 25% (above 65.5 percentiles) of the distribution. The results under other definitions of low and high recommendations are qualitatively similar.

**Result 5**. Human monitors are less likely to adjust recommendations that contain larger errors and, if they do so, correct them less than those with smaller errors. Participants tend to intervene less with very high or very low recommendations.

### Selection

Our design allows to see if participants who chose a certain source of recommendations are better able to monitor its recommendations as compared to those who were exogenously assigned to it. Our results do not offer strong evidence of a selection. Participants might be more likely to adjust a recommendation from an “imposed” source (Chosen 62% and Imposed 65%, ttest *p* = 0.07). Yet, this result is not robust (see S4 Table in S1 File). Furthermore, there is no difference in the size of the adjustments or their accuracy if we compare those who selected themselves to receive a certain type of recommendations and those who were exogenously assigned (S4 Table in S1 File, specifications 3 and 4).

**Result 6**. We do not observe that participants are better able to monitor the recommendations stemming from their preferred source.

### General attitudes

As we conduct our experiment at the leading technical university, our sample might be believed to be more technology friendly than general population. Our participants generally report to be positive towards algorithms and statistical models. On the scale from 0 (strongly negative) to 100 (strongly positive) average answer is 67 with the distribution skewed to the right (S2 Fig in S1 File). Yet, even in this sample the attitudes towards using algorithms to make economic, legal or other important decisions that may affect a human are mixed with an average reply of 50 and almost uniform distribution along the scale (S3 Fig in S1 File). Regardless of their attitudes towards the use of algorithms for such decisions, even our technology friendly participants are almost unanimous that a human needs to always remain in the loop (0 never, 100 always, average 75.3 with over 30% of participants choosing 100 and 50% of participants choosing values larger than 85, S4 Fig in S1 File). However, as our results show, while Human-in-the-Loop increases acceptance of algorithmic recommendations, as such it does not guarantee neither higher accuracy of decisions on average nor avoiding “extreme” mistakes.

**Result 7**. Our sample is in general technology friendly and technology-savvy, yet the majority is of the opinion that that algorithms used for important decision-making should (almost) always be monitored by a human.

## Discussion and conclusion

Automated decision-making can exist in many variations, from a full delegation of the decision to the automated agent to the human staying in the loop. This study considers how such differences in the distribution of decision authority between humans and automated decision supports affect the human principals’ willingness to use and engage with them. It has also considered the effects on decision accuracy, highlighting a potential trade-off.

In more details, and as the first main finding, our experiment documents a widespread willingness to use automated decision supports—that is, in contrast to some previous studies, our experiment fails to document algorithm aversion. Indeed, when given the choice, the majority of participants across all treatments prefers to receive an estimate produced by an algorithm over the one produced by an equally well-performing human. This preference for algorithmic recommendations becomes even stronger if participant can remain in the decision loop to monitor or intervene. If allowed to adjust the recommendation, participants are also more confident in the resulting decisions regardless of the source of the recommendation. We hence find that people are not algorithm-averse, in particular when the automated decision is construed with a human-in-the-loop. In line with public opinion surveys [25, 26], a clear majority of participants thinks humans should remain involved in automated decision-making when these decisions are legally, economically or similarly important.

However, we also find that when the human monitors are allowed to adjust recommendations in such a Human-in-the-Loop setup, the accuracy of the performance predictions decreases. Participants are even found to be less likely to intervene when the errors in the recommendations are larger—and, if they do intervene, they correct these larger errors by less. In other words, if the main motivation of putting a human in loop is quality control, human monitors seem to fail at their task. Yet, as stated above, our participants state a clear preference for keeping human monitors—either revealing that they are unaware of this accuracy reduction or showing that they are willing to forego some decision quality in return for keeping human oversight. Future studies to investigate this further could prove insightful.

### Limitations

When interpreting the results, two caveats deserve special attention: First, for the purpose of the experiment, the recommendations from the other human/the algorithm were curated to be equally accurate, both on average and for each iteration of the task. In reality, ample empirical evidence suggests algorithms generally make better forecasts than humans [e.g., 32, 33, 35]. In our experiment participants who chose a human recommendation did not necessarily make less accurate final performance predictions and therefore lower uptake of the algorithmic recommendations did not affect the quality of the decisions.

Second, in our experiment human interventions decreased accuracy. Accuracy of adjustments may depend on the expertise of human monitors and information available to them. Regarding the former point, one may argue that engaging experts as monitors would improve the quality of human interventions. Yet, based on the previous literature it also seems sensible to suggest that our participants may have relied on the recommendations more than experts would have, thus improving their accuracy [e.g., 11]. On the latter point, our experiment tested an environment where human monitors had access to and could process the same information as the algorithm. In a more sophisticated system, the number of features incorporated into the automated recommendation may exceed human capacity. If this is the case, human monitors may have to rely on inferior (or at least limited) information when deciding on the adjustment or may be overwhelmed if all features included by the algorithm are revealed in an attempt to make the system more explainable [36]. This imbalance may increase the risk of decreasing decision accuracy due to human intervention further.

For the discussion of how to best design environments that involve automated decision-making (e.g., the EU’s General Data Protection Regulation, EU’s Artificial Intelligence Act, or the Council of Europe’s discussion on a potential AI convention) our results may be informative as they point to a trade-off: the experiment shows a case where the retention of human involvement may improve the uptake of algorithmic recommendations, yet decrease accuracy of the final decisions. The resolution of this trade-off is bound to be context-specific and depend, among others, on the decision at hand, the performance and reliability of automated supports and human monitors. Yet, our results demonstrate that including a human-in-the-loop as a default for all hybrid decisions might not be desirable. More generally, our findings show the need for careful consideration of the distribution of agency in automated decision-making situations.

## Supporting information

S1 File (ZIP)
