# Supplementary material for: Putting a human in the loop: Increasing uptake, but decreasing accuracy of automated decision-making
Source: PLoS One. 2024 Feb 9;19(2):e0298037. doi: 10.1371/journal.pone.0298037 (PMC10857587; doi:10.1371/journal.pone.0298037)
Supplement: S1 File — (ZIP) [file pone.0298037.s001.zip › Supporting information S1 & S2.pdf]

S1 Additional Tables and Figures

S1.1 Additional Figures

Fig S1. Accuracy measured as an absolute deviation between a submitted estimation and a true performance percentile. A smaller deviation corresponds to higher accuracy of the prediction. In Delegation treatment it reflects accuracy of the received recommendation itself as participants could not adjust it. 95% confidence intervals.

Fig S2. What is your general attitude towards algorithms or statistical models? The vertical line represents average response. The question appeared in the post-experimental questionnaire.

Fig S3. How acceptable do you find the use of statistical models or algorithms to make economic, legal or other important decisions that may affect a human? The vertical line represents average response. The question appeared in the post-experimental questionnaire.

Fig S4. If a statistical model or an algorithm were to be used to make an economic or legal decision that affects a human, how often do you think a human should remain involved to oversee this statistical model or algorithm? The vertical line represents average response. The question appeared in the post-experimental questionnaire.

Fig S5. Number of times (out of 4 possible choices) participants chose to receive recommendations by an algorithm across both treatments.

Fig S6. Number of times (out of 4 possible choices) participants chose to receive recommendations by an algorithm. Percentages calculated by treatment.

S1.2 Additional Tables

Table S1. Marginal effects of the probit estimation. Demographic controls include age, gender and if participant studies a STEM discipline or social sciences as dummy variables.

Table S2. In model (1) the decisions of 6 participants (120 decisions) were omitted becasue of all positive or all negative outcomes. In models (2) and (3) standard errors are clustered at individual level.

Table S3. Likelihood to adjust the recommendation and accuracy by features of the profile and the recommendation. Specification (2) is conditional on non-zero adjustment. High and low recommendations are classified as those in the lowest 25% (below 42 percentile) and highest 25% (above 65.5 percentile) of the distribution.

Table S4. Selection: Adjustment of recommendations if the choice of the preferred source was followed or reverted. Models (1) and (2) omit decisions of 6 participants (120 obs decisions) because of all positive or all negative outcomes. Models (3) is conditional on adjusting the received recommendation.

S2 Materials: Excerpt

Fig S7. Example Screen. Delegation condition. Submission of an estimate.

Fig S8. Example Screen. Human-in-the-Loop condition. Submission of an estimate. Clicking on "No, amed" opens a new field.

Fig S9. An excerpt of experimental instructions. Description of a statistical model and another participant.
