# Supplementary figures and images for: Putting a human in the loop: Increasing uptake, but decreasing accuracy of automated decision-making
Source: PLoS One. 2024 Feb 9;19(2):e0298037. doi: 10.1371/journal.pone.0298037 (PMC10857587; doi:10.1371/journal.pone.0298037)

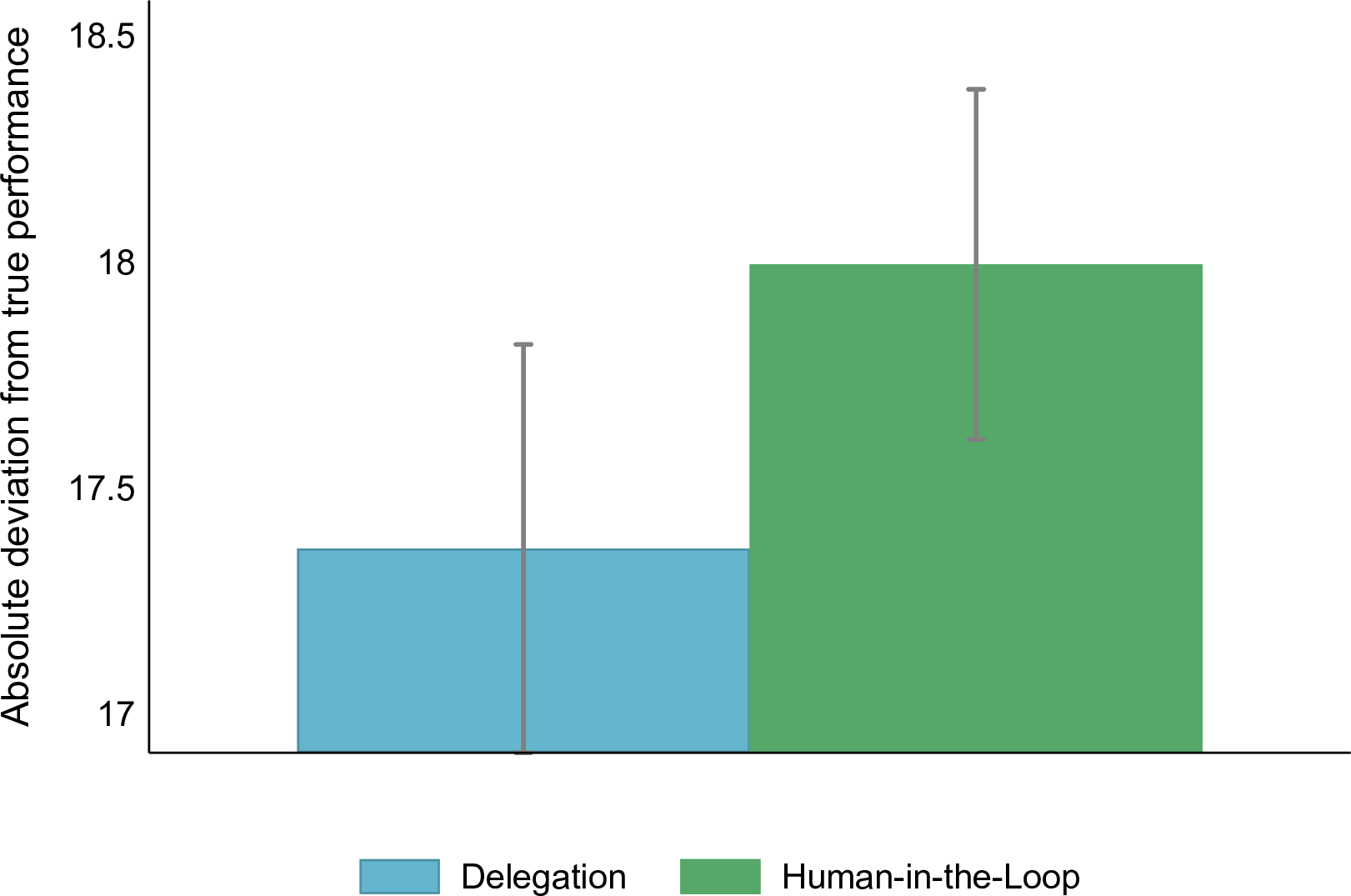

Supplement: S1 File — (ZIP) [file pone.0298037.s001.zip › FigS1.tif]

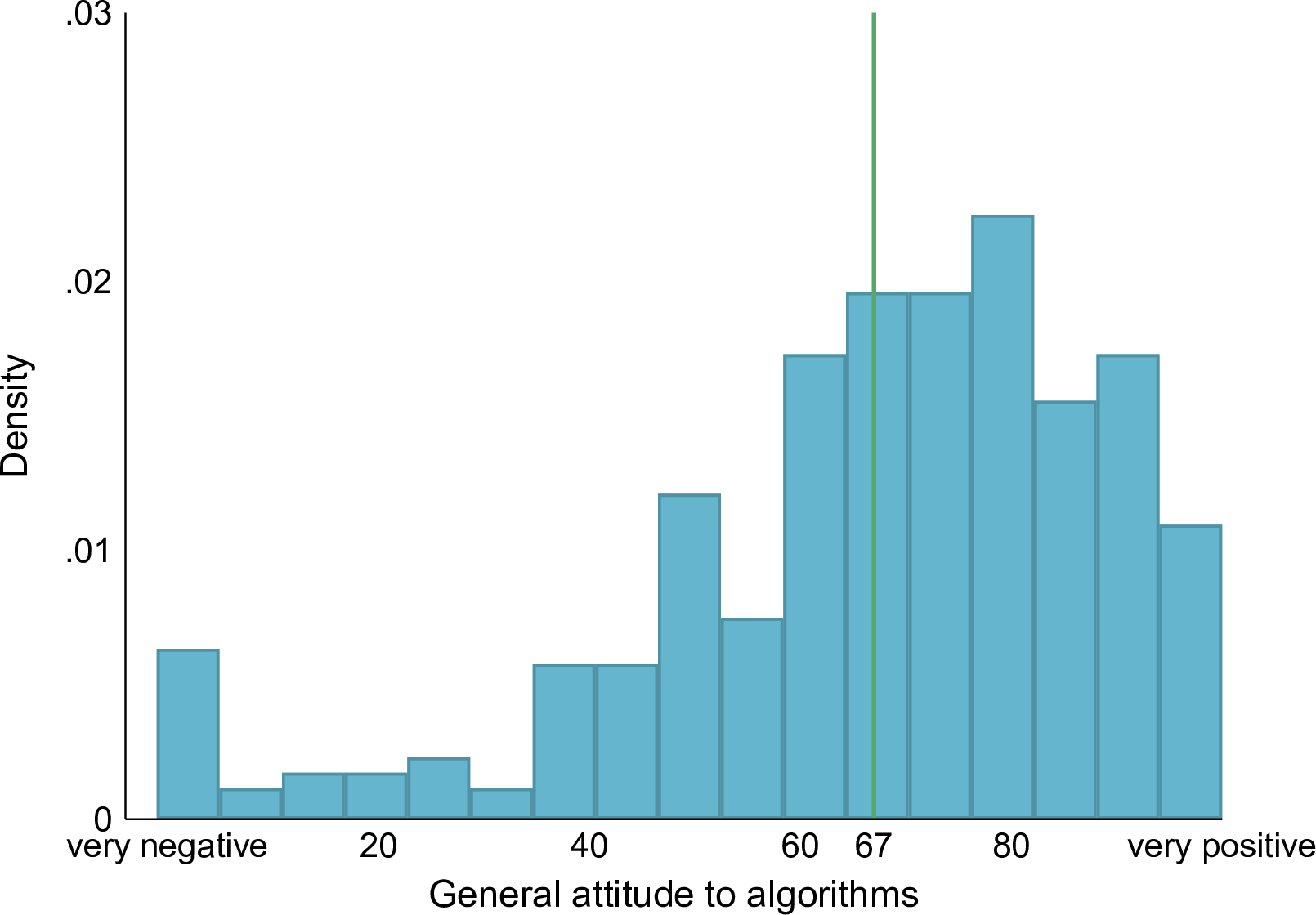

Supplement: S1 File — (ZIP) [file pone.0298037.s001.zip › FigS2.tif]

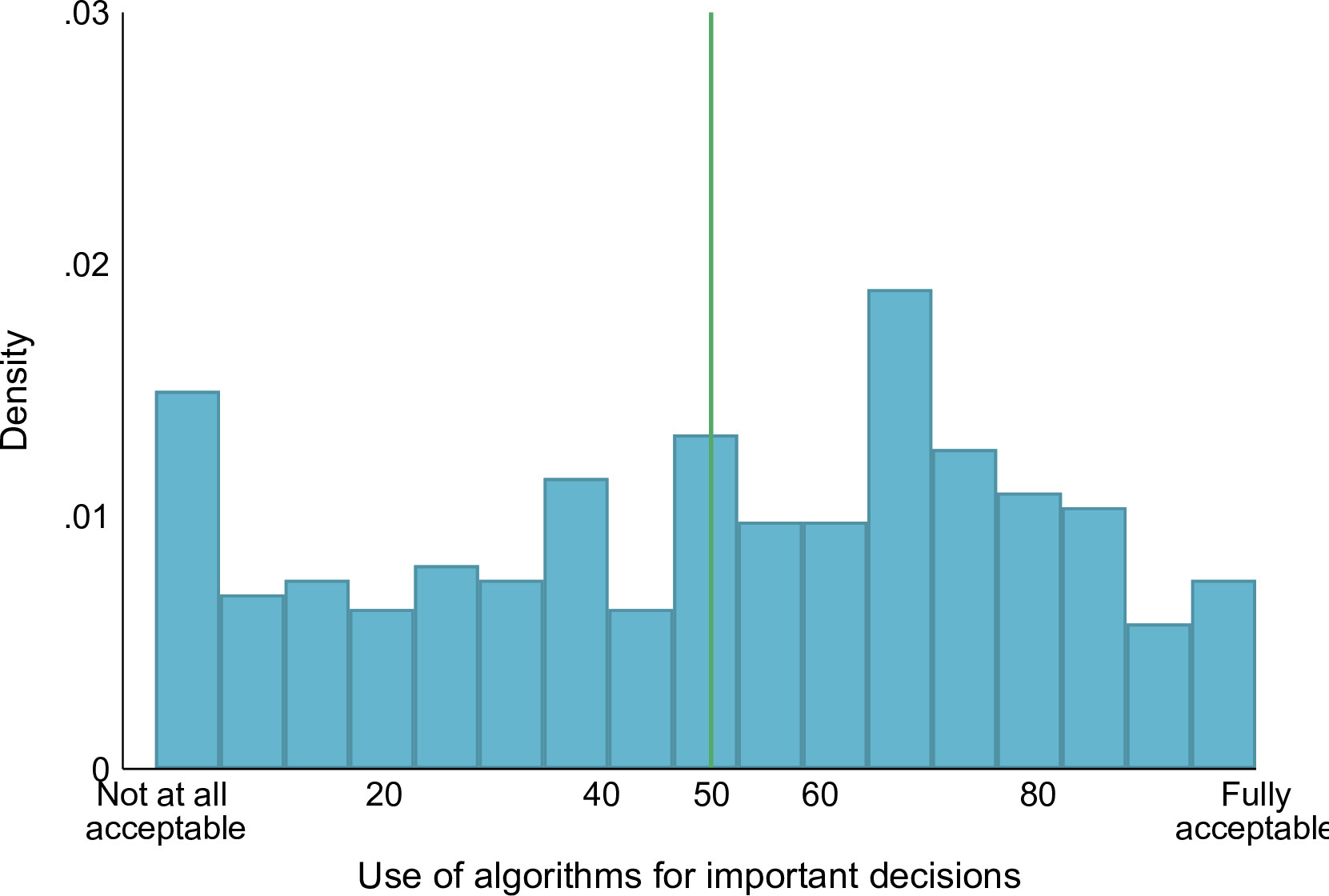

Supplement: S1 File — (ZIP) [file pone.0298037.s001.zip › FigS3.tif]

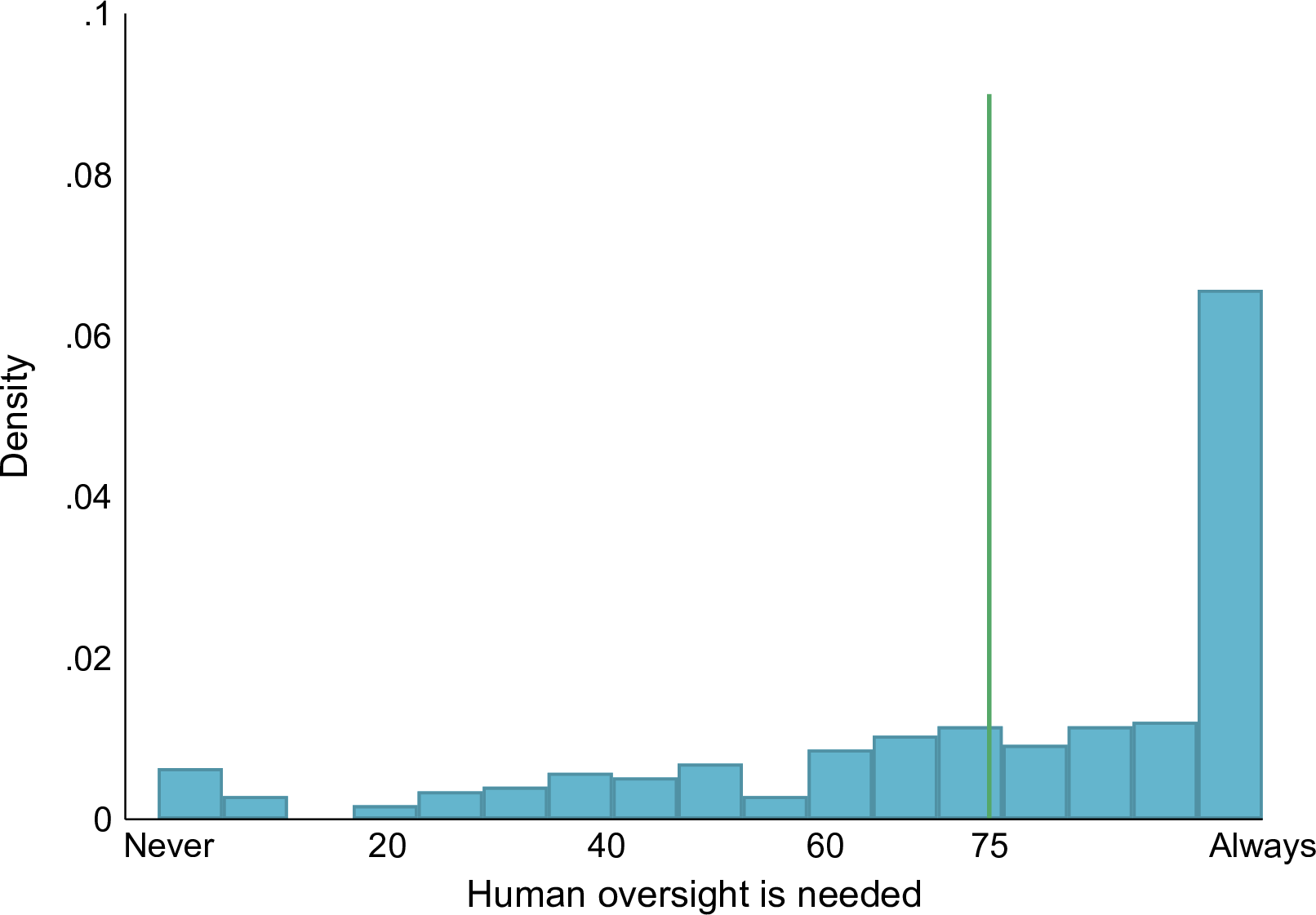

Supplement: S1 File — (ZIP) [file pone.0298037.s001.zip › FigS4.tif]

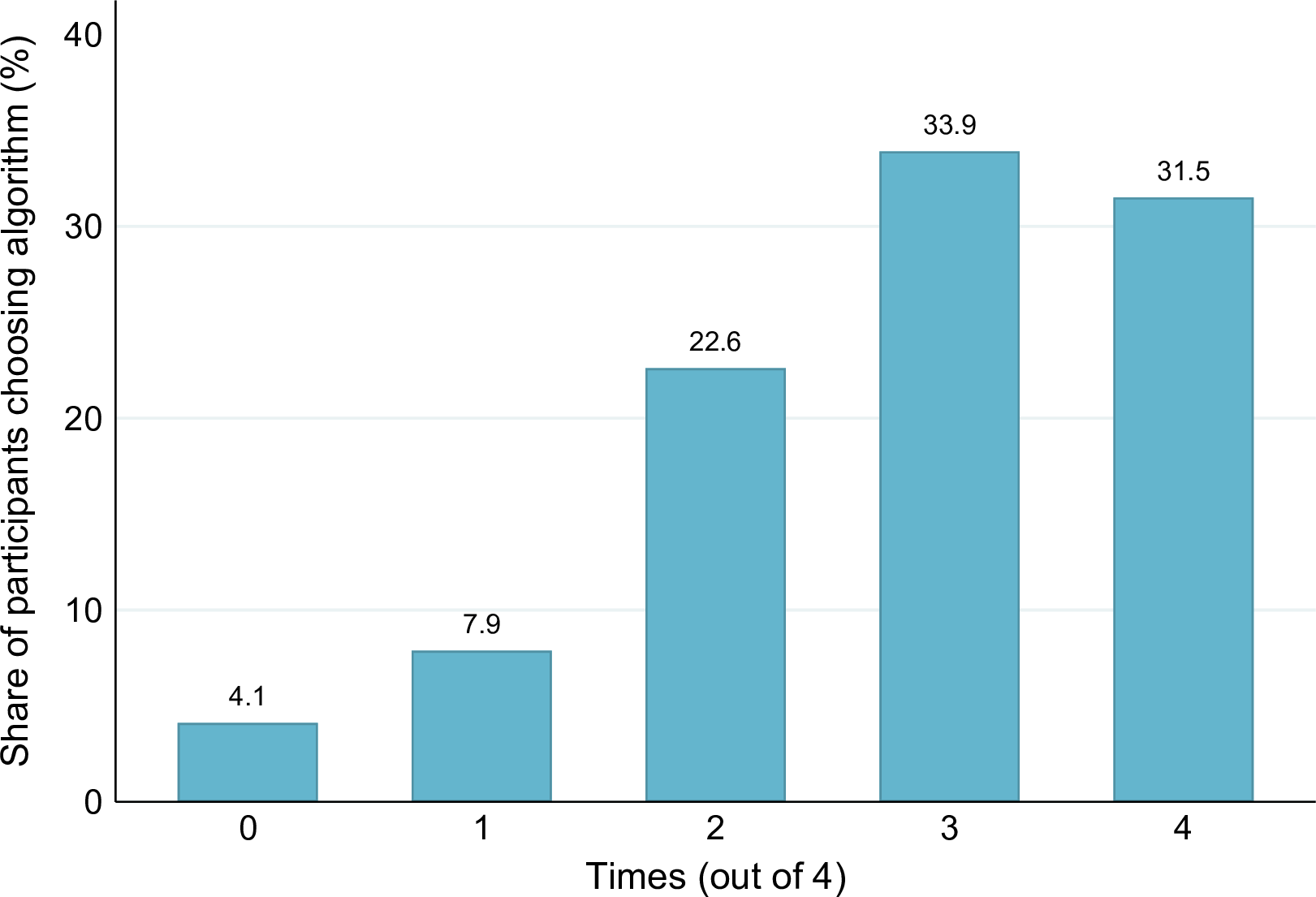

Supplement: S1 File — (ZIP) [file pone.0298037.s001.zip › FigS5.tif]

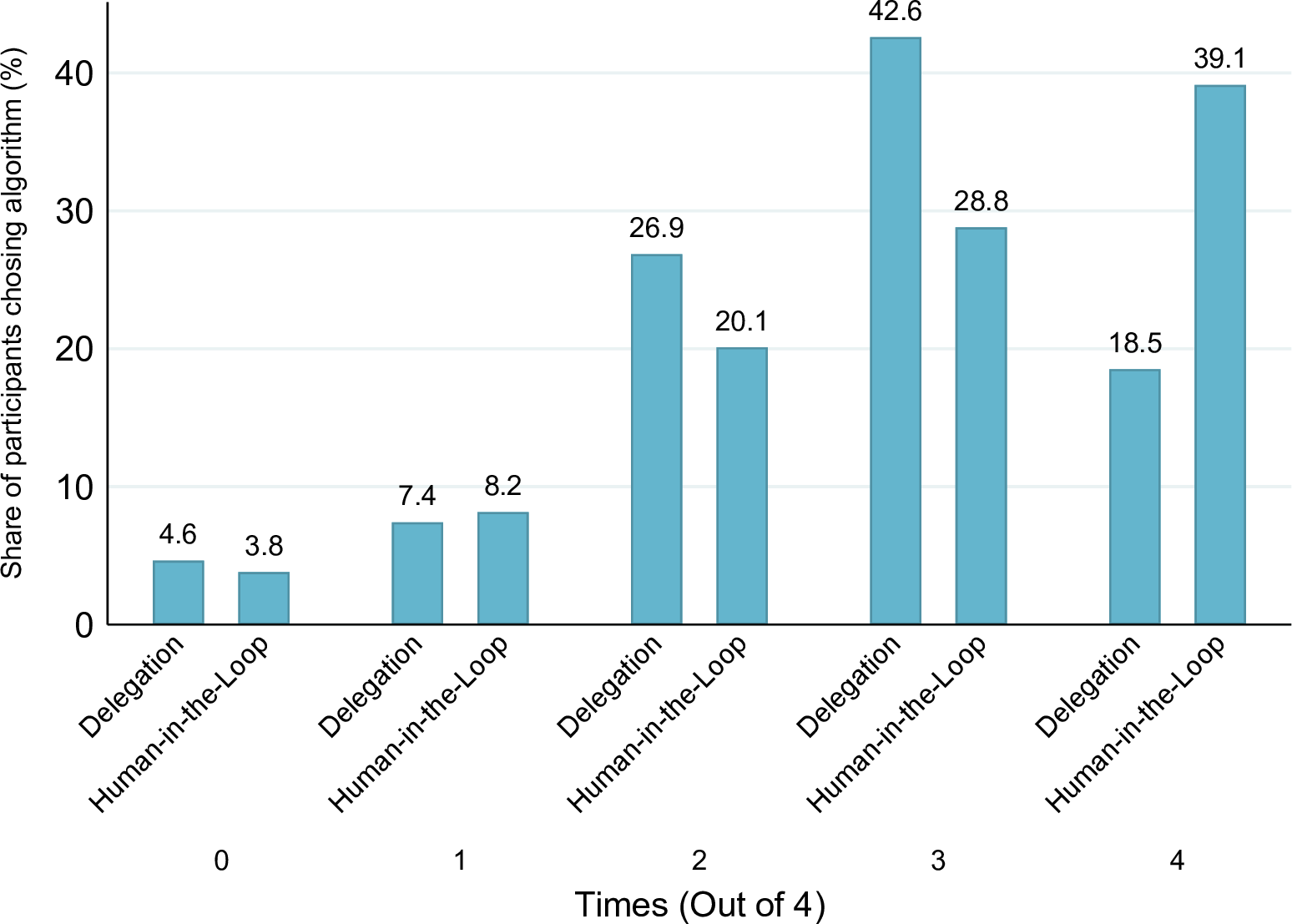

Supplement: S1 File — (ZIP) [file pone.0298037.s001.zip › FigS6.tif]

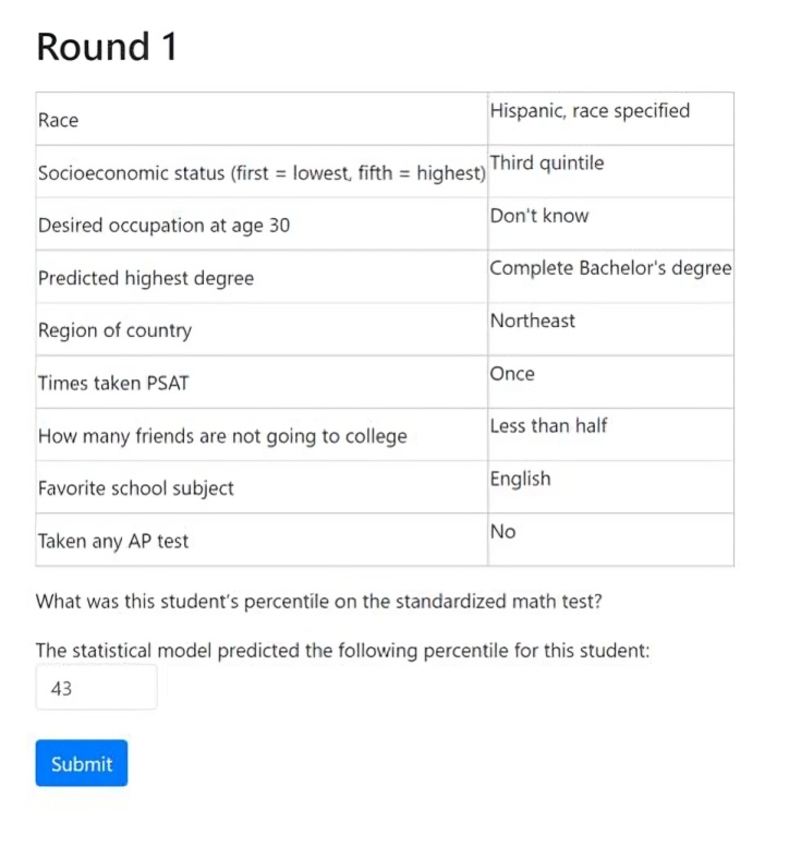

Supplement: S1 File — (ZIP) [file pone.0298037.s001.zip › FigS7.tif]

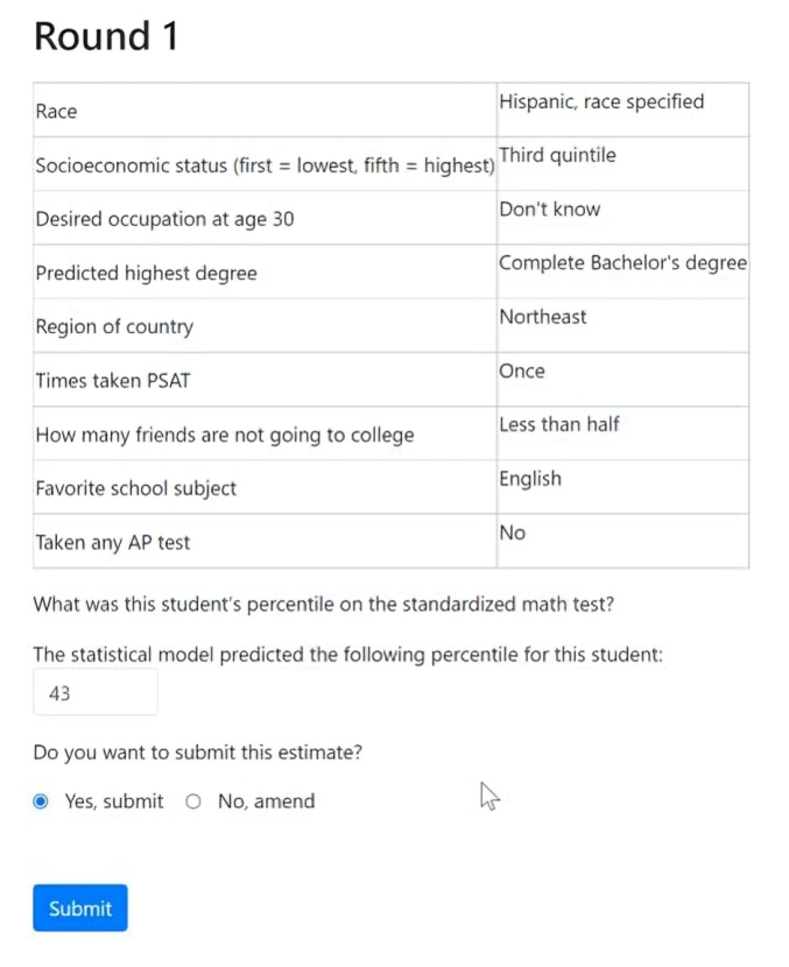

Supplement: S1 File — (ZIP) [file pone.0298037.s001.zip › FigS8.tif]

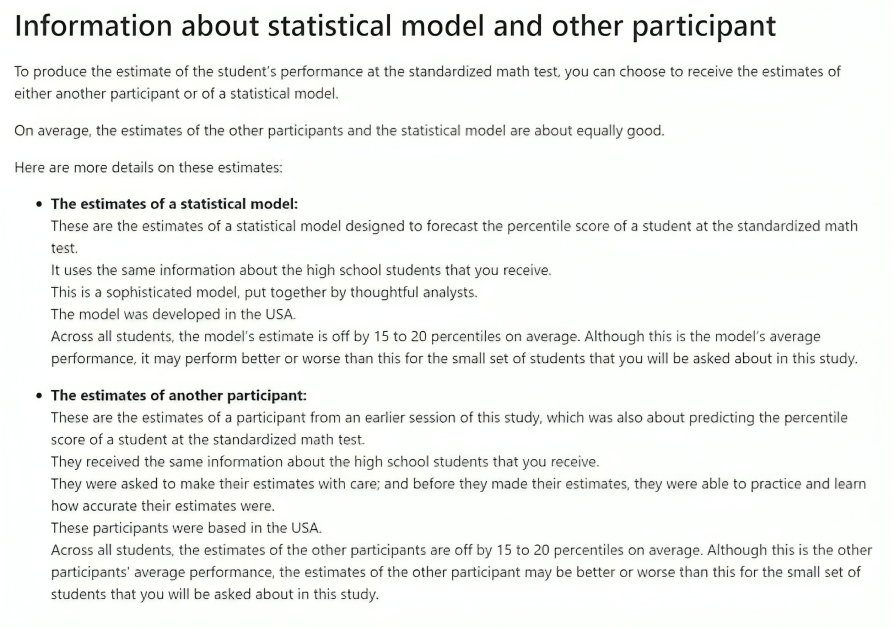

Supplement: S1 File — (ZIP) [file pone.0298037.s001.zip › FigS9.tif]
